# Doppler ultrasound cardiac gating of intracranial flow at 7T

**DOI:** 10.1186/s12880-020-00523-x

**Published:** 2020-12-09

**Authors:** Karin Markenroth Bloch, Fabian Kording, Johannes Töger

**Affiliations:** 1 The Swedish National 7T Facility, Lund University Bioimaging Center, Lund University, Klinikgatan 32, BMC D11, 22242 Lund, Sweden; 2 Department of Diagnostic and Interventional Radiology and Nuclear Medicine, University Medical Center Hamburg- Eppendorf, Hamburg, Germany; 3 Northh Medical GmbH, Röntgenstraße 24, 22335 Hamburg, Germany; 4 Diagnostic Radiology, Department of Clinical Sciences Lund, Lund University and Skane University Hospital Lund, Lund, Sweden

**Keywords:** Ultra-high field MRI, Flow quantification, Cardiac synchronization, Neurovascular, Doppler ultrasound, ECG

## Abstract

**Background:**

Ultra-high field magnetic resonance imaging (MR) may be used to improve intracranial blood flow measurements. However, standard cardiac synchronization methods tend to fail at ultra-high field MR. Therefore, this study aims to investigate an alternative synchronization technique using Doppler ultrasound.

**Methods:**

Healthy subjects (n = 9) were examined with 7T MR. Flow was measured in the M1-branch of the middle cerebral artery (MCA) and in the cerebral aqueduct (CA) using through-plane phase contrast (2D flow). Flow in the circle of Willis was measured with three-dimensional, three-directional phase contrast (4D flow). Scans were gated with Doppler ultrasound (DUS) and electrocardiogram (ECG), and pulse oximetry data (POX) was collected simultaneously. False negative and false positive trigger events were counted for ECG, DUS and POX, and quantitative flow measures were compared.

**Results:**

There were fewer false positive triggers for DUS compared to ECG (5.3 ± 11 vs. 25 ± 31, *p* = 0.031), while no other measured parameters differed significantly. Net blood flow in M1 was similar between DUS and ECG for 2D flow (1.5 ± 0.39 vs. 1.6 ± 0.41, bias ± 1.96SD: − 0.021 ± 0.36) and 4D flow (1.8 ± 0.48 vs. 9 ± 0.59, bias ± 1.96SD: − 0.086 ± 0.57 ml). Net CSF flow per heart beat in the CA was also similar for DUS and ECG (3.6 ± 2.1 vs. 3.0 ± 5.8, bias ± 1.96SD: 0.61 ± 13.6 μl).

**Conclusion:**

Gating with DUS produced fewer false trigger events than using ECG, with similar quantitative flow values. DUS gating is a promising technique for cardiac synchronization at 7T.

## Background

For many magnetic resonance imaging (MRI) applications, it is necessary to synchronize the MR data acquisition to the cardiac rhythm, either to investigate motion due to the heartbeat, or to avoid artifacts or image blurring caused by the pulsation. Examples relevant for MRI neuroimaging at 7T are neurovascular flow and velocity mapping [1–3], arterial pulsation measurements [4] and cardiac gated functional magnetic resonance imaging (fMRI). In clinical practice, cardiac synchronization is done with electrocardiogram (ECG) or pulse oximetry (POX), techniques that through continuous technical development work well at both 1.5 T and 3 T [5]. However, at high field strengths such as 7T, ECG-based methods become unreliable due to the magnetohydrodynamic effect (MHD) [6, 7]. Several studies find that ECG gating at 7T fails in 10–20% of examinations, and is suboptimal in another 20–30%, leading to prolonged scan time or reacquisitions [8–12]. POX synchronization can be disturbed by certain gradient schemes and is sensitive to hand motion and skin temperature [13]. In addition, the trigger signal from POX is delayed 200–300 ms compared an ECG trigger at the R-peak, and the POX trigger has a higher temporal variability (jitter) due to pulse wave broadening. This can cause image blurring and might not be acceptable when high temporal resolution is required [14, 15].

Development of reliable cardiac synchronization techniques is therefore essential for cardiac-gated examinations to benefit from ultra-high field MR. Several new methods for cardiac synchronization have been proposed, such as self-gating [16–18], advanced ECG processing [9, 19], scattering of a parallel transmit RF coil [20, 21] or using acoustic [14, 22], optical [23] or magnetohydrodynamic (MHD) signals [23]. However, these methods require hard- or software that are difficult to implement or not readily available.

At 7T, accurate high-resolution flow quantification in small vessels is possible, provided that an accurate and reliable gating method is applied. This work investigates the efficacy of a synchronization technique based on Doppler ultrasound (DUS) for the application of intracranial velocity mapping, and compare it to vector ECG and POX. Gating with the DUS technique is expected to be beneficial at 7T, since it is not sensitive to MHD effects or magnetic field gradients. The feasibility of gating MR-scans by DUS was first investigated by Rubin et al. [24]. The safety and efficacy of DUS-gating at 7T has been investigated for cardiac imaging [25]. In more recent work, DUS-gating was shown to be as successful as ECG for cardiac cine imaging at 1.5 T [15], cine imaging and phase contrast imaging at 3 T [26, 27], and fetal cardiac imaging [28–30]. The flow in small vessels in the brain, including pathways for cerebrospinal fluid, are orders of magnitude smaller than for cardiovascular applications.

Therefore, purpose of this paper is to investigate the efficacy of a DUS device for gating of intracranial flow measurements at 7T. Specifically, the aim is to quantitatively compare false positive and negative trigger events as well as the results of quantitative flow measurements when using DUS and ECG, respectively.

## Methods

### Population

Healthy volunteers (n = 9, age range 22–45 years, BMI range 21–31, two female) without cardiovascular disease were enrolled in the study. The local Ethical Review Board approved the study, and all subjects provided written informed consent.

### Doppler ultrasound for cardiac synchronization

The Doppler ultrasound (DUS) device and signal processing algorithms used in this work consist of a custom-built DUS unit and a signal processing unit [15, 25, 26] (north medical GmbH, Hamburg, Germany). The DUS unit transmits 1 MHz pulses with a repetition frequency of 3.2 kHz to a single 1 × 1 cm-piezoelectric element transducer, made from non-magnetic lead zirconate titanate, placed on the subjects’ chest. The long single conductive line is shielded with copper, 3 mm in diameter, and six RF/cable traps are placed with 10 cm distance to avoid coupling to the electric field (E-field). In addition, a resonant circuit is placed at the end of the transmission line. A safety assessment was performed in [25] for the case where the transducer was placed within the transmitting volume of a 7T cardiac array coil. In the present work, the transducer is placed more than 25 cm from the end of the transmitting head coil, where the E- and H-fields are significantly reduced compared to the placement in [25]. In this study, B_1_ mapping was performed in a phantom setup prior to any human examinations, and neither the transducer nor the cable affected the B_1_ field [25]. The DUS processing unit was placed inside the scanner room, keeping it at a distance corresponding to a field of 20 mT (200 Gauss) following local safely guidelines for investigational devices. The DUS signal processing algorithms were implemented on a microcontroller (STM32F4, STMicroelectronics, Geneva, Switzerland). An important feature of the processing is to remove the ultrasound signals originating from blood flow, while keeping the dominant component from wall motion [31]. A peak detection algorithm (previously described in detail [15]) generated the 5 V TTL trigger signal, which was fed into the MR system via the standard ECG input. In brief, after low-pass filtering, peaks within the DUS signal were found using a discrete wavelet transform. As the ultrasound signal reflects the motion of both blood and myocardium, the appearance of the signal can vary. In order to restrict the data to be analyzed in real time, a window of interest is created where the next heartbeat is anticipated. A continuous autocorrelation of the first 2.5 s of the DUS signal is used to estimate the mean RR-interval and to create a window of interest. The peak detection algorithm is restricted to the data in the window of interest, and the best fitting peak within this window is used to generate a trigger pulse.

The DUS efficacy was compared to the vendor’s 4-lead vector ECG system, using a fiber optical connection to the scanner and the vendor provided detection algorithms [5]. As a common reference, the vendor’s pulse oximetry (POX) system was used during both DUS- and ECG gated examinations. As the DUS device used the ECG system’s input connections, trigger signals from ECG and DUS could not be recorded simultaneously in the present setup.

**Fig. 1:**
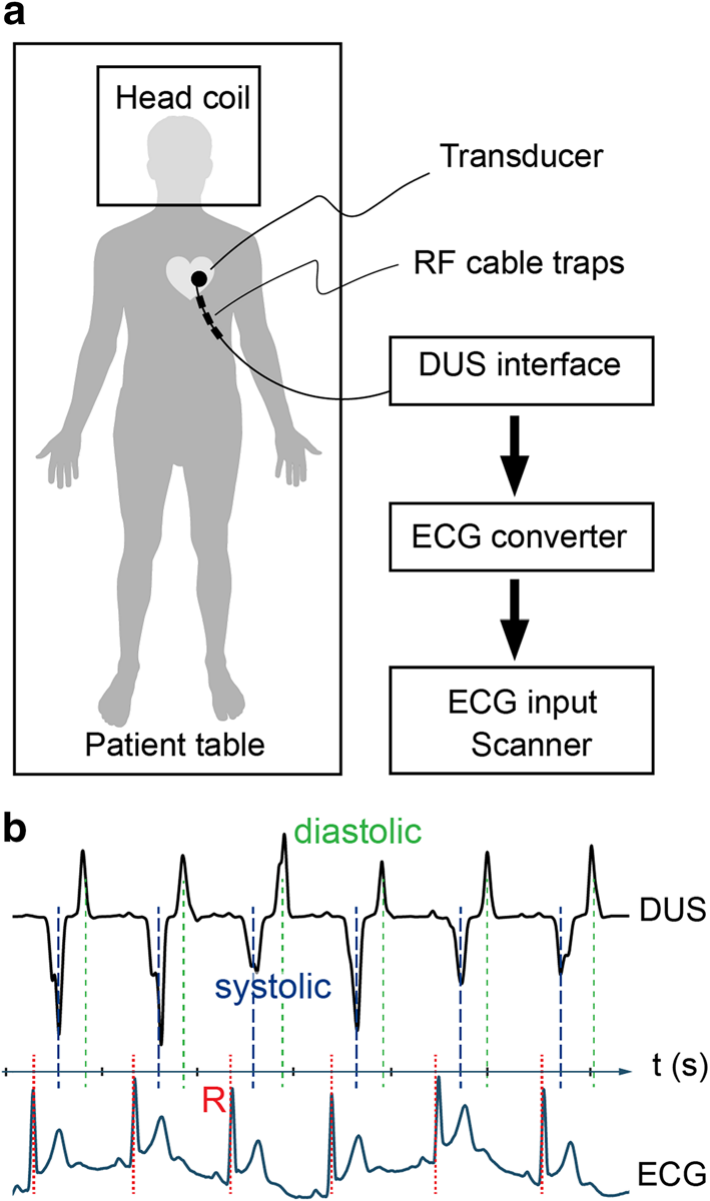
**a** Schematic view of the Doppler ultrasound (DUS) setup. The DUS transducer was placed over the heart on the subject’s chest. The exact location was adjusted while observing the signal on the DUS interface box to achieve a clear signal. The trigger signal from the DUS box was then converted to electrocardiogram (ECG) levels and connected to the ECG input device on the scanner. The cable between the transducer and DUS interface box included several radiofrequency (RF) traps to avoid heating of the cable. **b** Example of the timing of the triggers from the DUS diastolic wave (green, short dashes), DUS systolic wave (blue, long dashes) and ECG R-wave (red, dotted). As seen on the time scale, the triggers occur at different point in the cardiac cycle

Placement of the DUS sensor on the subjects’ chest was performed with visual feedback of the raw DUS signal, provided on a display on the signal processing unit (Fig. 1a). The sensor was placed at the sternum and moved towards the left side and towards the head until a strong signal was displayed on the screen. The sensor was then fastened with surgical tape and a strap around the subjects’ chest (Fig. 1a). The time for the setup was comparable to that for the ECG (a few minutes per subject). The DUS signal is the results of both myocardial motion and blood flow, and assigning standard echocardiography waves to the signals can be misleading. The DUS transducer picks up two peaks suitable for triggering; one systolic peak and one diastolic peak.

### MR data acquisition

All examinations were performed using an actively shielded 7T MR scanner (Achieva Philips, Best, the Netherlands) and a dual channel transmit head coil with 32 receive elements (NOVA Medical, Wilmington, MA, USA). Retrospectively cardiac gated quantitative velocity mapping was performed both with through-plane phase contrast (2D flow) and three-dimensional, three-directional phase contrast (4D flow). The 2D flow measurements were used to assess blood flow in the right M1 branch of the middle cerebral artery (MCA) and flow of cerebrospinal fluid (CSF) in the cerebral aqueduct (CA). Additionally, flow in both branches of the M1 were also quantified with 4D flow. Scan parameters for 2D flow measurements of blood and CSF were the same, except for the velocity encoding sensitivity (v_enc_) (150 cm/s and 15 cm/s for the M1 and CA, respectively) and the inplane resolution (0.5 × 0.5 mm^2^ and 0.3 × 0.3 mm^2^, respectively). To keep the temporal resolution comparable, the number of cardiac phases was maximized in each individual. Scan parameters for the 2D and 4D flow sequences are listed in Table 1.

**Table 1:** Acquisition parameters of the 2D and 4D flow scans

	2D Flow M1R^a^	2D Flow CA^b^	4D Flow CoW^c^
Scan duration (min:s)	1:08–1:51	1:40–3:04	4:34–7:27
Heart rate (median)	50–72 (66)	50–79 (66)	51–84 (65)
Resolution (mm × mm)	0.5 × 0.5	0.3 × 0.3	0.7 × 0.7
Slice thickness (mm)	5.0	5.0	0.7
Time frames (median)	14–20 (17)	13–20 (20)	6–10 (7)
FOV (mm × mm)	200 × 220	200 × 220	180 × 180 × 21
TR (ms)	10	10	5.6
TE (ms)	2.8	4.3	2.8
α (°)	5	5	8
BW (Hz/pixel)	405	403	404
Turbofactor	2–3	2–3	6
SENSE	2.0	2.0	3.5
Temporal resolution (ms)	40–60	40–80	130–140
v_enc_ (cm/s)	150	15	150

^a^Right M1 branch of the middle cerebral artery (MCA)

^b^Cerebral aqueduct

^c^Circle of Willis

In addition to the velocity mapping scans, the protocol consisted of a B_0_-map for second order image-based shimming and a 3D T1-weighted fast gradient echo scan for positioning of subsequent scans (1 mm^3^ isotropic resolution).

Each scan session consisted of two parts. The subject was equipped with sensors for the ECG (standard MR electrodes) or the DUS transducer. The MR scanners pulse oximeter (POX) was placed on the subjects left middle finger. The protocol was executed, after which the patient table was moved out of the scanner to allow for exchange of the sensors, keeping the subject's head in the same position. The table was moved back into the bore, and the same set of sequences were performed. To minimize possible systematic errors due to the order, five subjects started with the DUS setup and four with the ECG setup. All trigger signals as well as POX and ECG waveforms were recorded in a log file. The POX signals were acquired simultaneously with the DUS/ECG gating signal, but was not used for gating.

### Analysis of trigger events

An in-house script (Matlab, MathWorks Inc., Natick, MA) extracted the detected trigger signals with time information for DUS/ECG and POX from the physiology log files for each scan, copied from the MRI scanner. The RR-intervals were deduced from the trigger signals, and a mean RR-interval (RR_mean_) was calculated for each scan. Three other measures were also deduced from the physiology log files; the total number of triggers, the number of triggers with an RR-interval larger than 1.5 × RR_mean_ (number of false positive triggers, FPT), the number of triggers with an RR-interval shorter than 0.6 × RR_mean_ (number of false negative triggers, FNT), and a combined sensitivity measure as defined in Eq. (1) [25].

1 $${\text{Sensitivity}} = 100 \times \left( {1 - \frac{(FPT + FNT)}{{{\text{Total}}\;{\text{ number}}\;{\text{ of}}\; {\text{triggers}}}}} \right)$$

FPT is thus the approximate number of erroneously detected triggers and FNT the number of missed true triggers. The definitions assume that the true heart rate variation is within the limits (0.6–1.5) × RR_mean._ This condition was fulfilled by setting the scanner to reject heart beats outside (0.7–1.3) × RR_mean_. Each subject had three gated scans for each triggering method (two 2D flow scans and one 4D flow scan). These three were combined into a total number of triggers, FPT and FNT per subject and trigger method.

### Analysis of quantitative flow data

The 2D flow data was analyzed in Segment 2.2 R6410 [32] (Medviso AB, Lund, Sweden). Phase background correction was performed by fitting a linear function to static tissue, defined by two ROIs semi-automatically placed in static tissue surrounding the vessel of interest. After subtraction of the phase background, the vessel of interest was manually delineated and velocity and flow parameters obtained.

The 4D flow data was analyzed in GTFlow R3.1.9 (Gyrotools LLC, Zürich, Switzerland). A linear function was fitted to static tissue as defined by thresholds set on velocity and magnitude. After this subtracting the linear fit of the phase background, vessels were delineated in a plane placed orthogonally to the M1 branches of the right and left MCA, and velocity and flow parameters were deduced. In healthy subjects, the flow in the two M1 MCA branches is on average be equal, even if it can differ in each individual [33]. Therefore, data from both the left and right M1 branches were used in the final 4D flow results.

### Statistical methods

*Trigger event data:* The measures of number of false positives (FPT), false negatives (FNT) and trigger sensitivity were compared with Wilcoxon matched-pairs test with *p* < 0.05 as the threshold for significance.

*MRI flow data:* Differences in measured flow and velocity values from ECG- and DUS gated scans were analyzed using Bland–Altman analysis, from which results are reported as bias ± 1.96 standard deviations (SD), corresponding to a 95% confidence interval (CI). For Wilcoxon’s matched pairs test, *p* < 0.05 was used as threshold for statistical significance.

## Results

For two subjects, the ECG signal was of so low quality that no signals were generated, and the comparative flow measurements were instead gated with the POX signal. For two other subjects, the ECG gated scans had to be reacquired between 1 and 5 times as the low quality of the ECG caused excessively long or aborted scans. The low quality of the ECG signal was reflected in both false negative and false positive triggers in the quantitative analysis. The range of heart rates over all subjects was 50–84 bpm.

For DUS, the synchronization was done on a systolic peak in five cases and on the diastolic peak in four cases. The timing difference between the triggers is shown in Fig. 1b. The DUS scans mainly suffered from false negative triggers which were to varying degree present in four of the subjects.

### Trigger event data

**Fig. 2:**
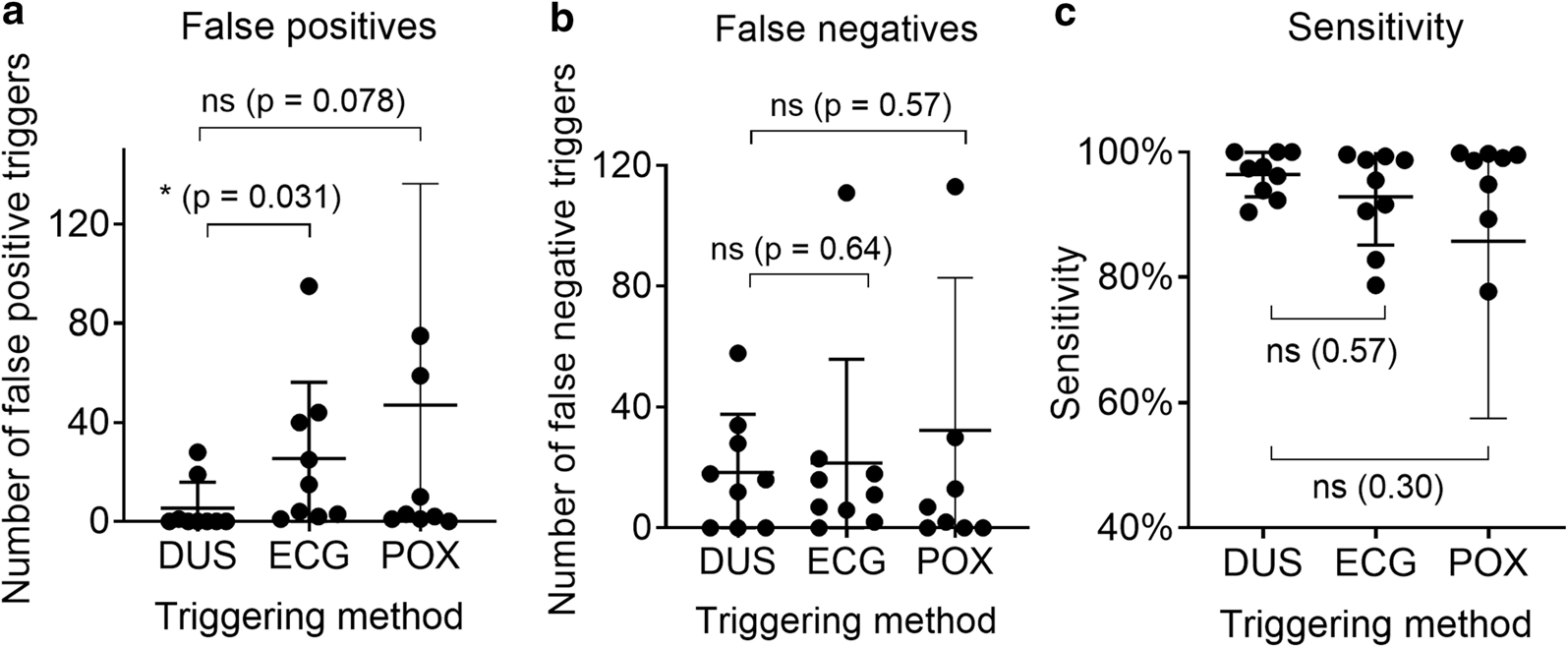
The graphs show the number of (**a**) false positive triggers, (**b**) false negative triggers and (c) the sensitivity measure for the DUS and ECG gated flow scans. The DUS and POX triggers are simultaneously acquired in the DUS triggered scans, and no gating is done on the POX signal

Figure 2 and Table 2 show a summary of the trigger event results. The number of false positive triggers (FPT) ranged between 0 and 28 (median: 0) for the DUS gated scans, 1 and 95 (median: 15) for the ECG scans and between 0 and 273 (median: 7) for the POX. The number of false negative triggers (FNT) was between 0 and 58 (median: 16) for DUS gated scans, 0 and 111 (median: 11) for the ECG scans and between 0 and 128 (median: 7) for the POX gating. The deduced sensitivity measure (Eq. 1) showed ranges of 90% to 100% (median: 97%) for DUS, and 79% to 100% (median: 95%) for EGC and 13 to 100 (median: 99) for POX gated scans. Only FPT showed a statistically significant difference between DUS and EGC gated scans (*p* = 0.031). All results are presented in Table 2.

**Table 2:** Summary of the trigger event results

	DUS		ECG		POX
False positive triggers					
Range (median)	0–28 (0)		1–95 (15)		0–273 (3)
Mean ± SD	5.3 ± 11		25 ± 31		47 ± 89
		*p* = 0.031(*)		*p* = 0.078 (ns)	
False negative triggers					
Range (median)	0–58 (16)		0–111 (11)		0–126 (7)
Mean ± SD	18 ± 19		22 ± 34		32 ± 50
		*p* = 0.64 (ns)		*p* = 0.57 (ns)	
Sensitivity (%)					
Range (median)	90–100 (97)		79–100 (95)		13–100 (99)
Mean ± SD	96 ± 3.5		93 ± 7.7		86 ± 28
		*p* = 0.37 (ns)		*p* = 0.30 (ns)	

As the DUS triggering was made in diastole in four subjects and in systole in five, we also investigated whether this made a difference. The analysis showed that there was no significant difference between the two (see Additional file 1: Fig. 1).

### Quantitative MR flow data

#### M1-branches of the MCA

**Fig. 3:**
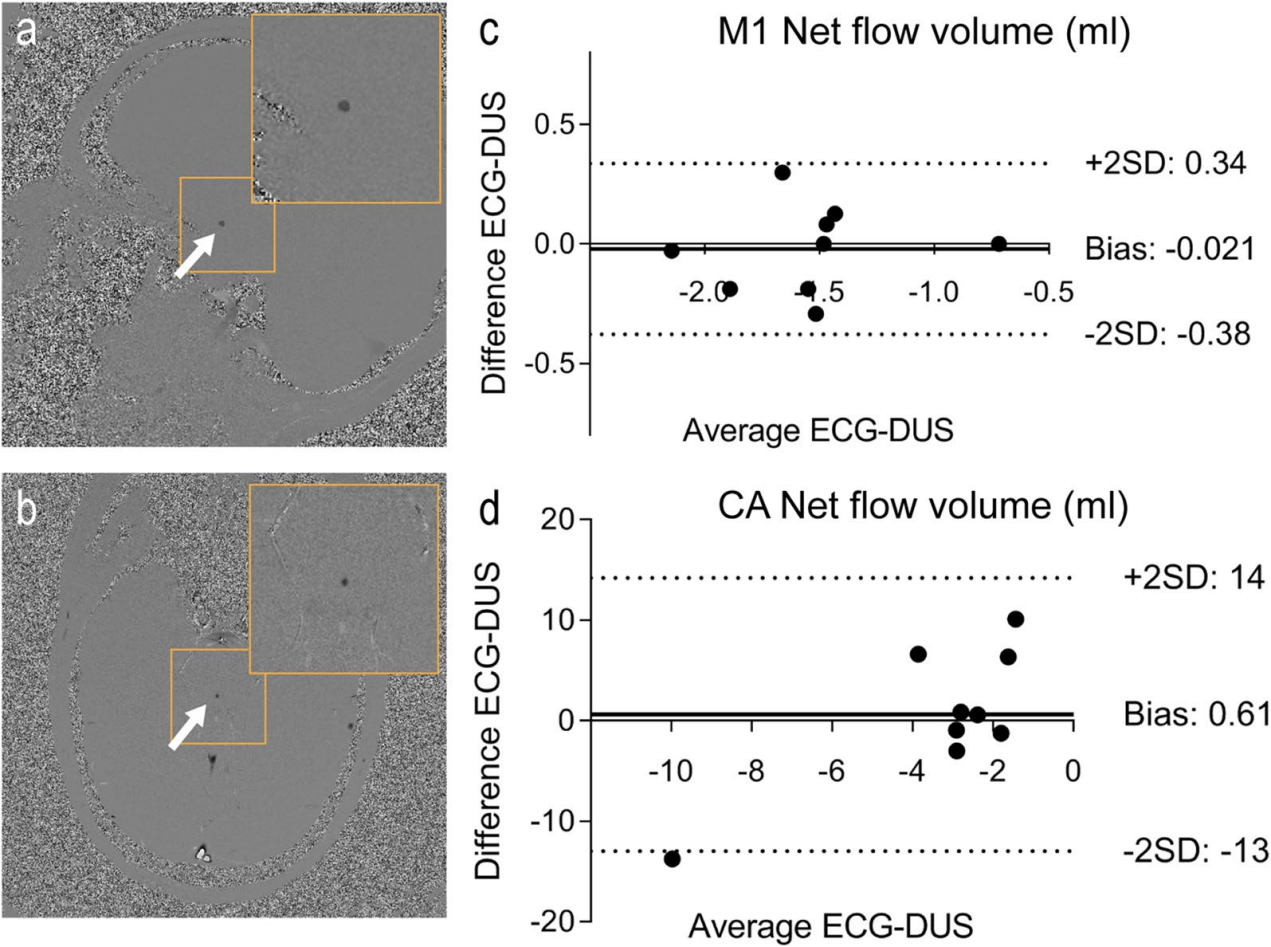
Examples of velocity maps for (**a**) the right M1 branch of the middle cerebral artery and (**b**) the cerebral aqueduct (CA), both gated with DUS. The right column shows Bland–Altman plots of the net volumes for (**b**) the right M1 MCA branch and (**d**) the cerebral aqueduct. The outlier in (**d**) is caused by misalignment of the measurement plane in one scans

Comparing the results from 2D flow gated by ECG and DUS in the right M1-branch of the MCA (M1R), the Bland–Altman results of net volumes, mean flow, mean velocity and peak velocity resulted in bias ± 1.96SD of − 0.021 ± 0.36 ml, − 0.010 ± 0.37 ml/s, 1.3 ± 3.9 cm/s and − 1.5 ± 108 cm/s respectively (Fig. 3, Table 3).

**Table 3:** Quantitative 2D flow results

	2D flow M1R^a^			2D flow CA^b^		
	ECG		DUS	ECG		DUS
Net flow volume						
(M1:ml, CA:μl)	1.6 ± 0.41		1.5 ± 0.39	− 3.0 ± 5.8		− 3.6 ± 2.1
Bias ± 1.96SD		− 0.021 ± 0.36			0.61 ± 13.6	
Mean flow						
(M1:ml/s, CA: μl /s)	1.7 ± 0.45		1.7 ± 0.41	− 3.0 ± 6.1		− 3.9 ± 2.4
Bias ± 1.96SD		− 0.010 ± 0.37			0.90 ± 15	
Mean velocity (cm/s)	27 ± 3.4		29 ± 4.3	− 0.078 ± 0.18		− 0.16 ± 0.13
Bias ± 1.96SD		1.3 ± 3.9			0.78 ± 5.4	
Peak velocity (cm/s)	90 ± 31		101 ± 30	− 9.7 ± 1.9		− 8.8 ± 2.9
Bias ± 1.96SD		− 1.57 ± 108			− 0.91 ± 4.7	

^a^Right M1 branch of the middle cerebral artery (MCA). Direction of positive flow is towards the right side of the patient, away from the Circle of Willis

^b^Cerebral aqueduct. Direction of positive flow is towards the ventricles

There were no statistically significant differences in any 2D flow measurements of the M1R branch of the MCA; as an example, the net flow volumes were 1.6 ± 0.4 ml/s vs. 1.5 ± 0.39 ml/s for ECG and DUS (*p* = 0.84) (results presented in Table 3).

**Fig. 4.**
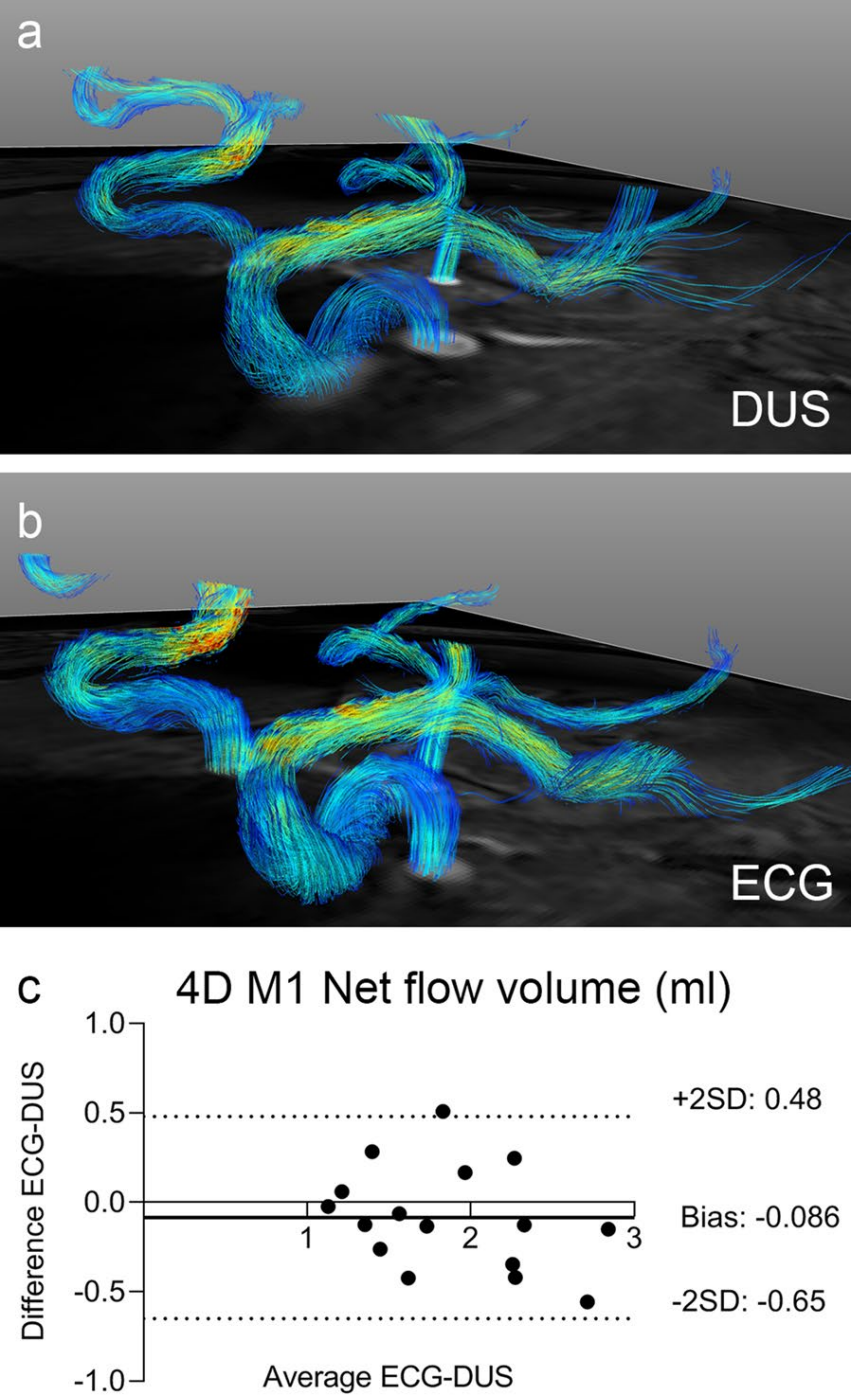
4D flow visualizations from one subject, gated with (**a**) DUS and (**b**) ECG. Panel (**c**) shows the Bland–Altman plots of the net volumes for left and right M1 MCA branches when comparing DUS and ECG gating. The difference in visualization is due to the positioning being slightly different between the two scans

Using 4D flow, the Bland–Altman analysis gave bias ± 1.96SD of − 0.086 ± 0.57 ml, − 0.071 ± 0.58 ml/s, − 0.077 ± 14 cm/s and 7.6 ± 44 cm/s for net volume, mean flow, mean velocity and peak velocity, respectively (Fig. 4, Table 4). There were no statistically significant differences; for example, the net flow volumes were 1.8 ± 0.48 ml and 1.9 ± 0.59 ml in the M1 for ECG and DUS (*p* = 0.84) (results presented in Table 4). For all M1 measurements, the positive direction of flow is outwards from the circle of Willis along the direction of flow.

**Table 4:** Quantitative 4D flow results

	4D flow M1^a^		
	ECG		DUS
Net volume (ml)	1.8 ± 0.48		1.9 ± 0.59
Bias ± 1.96SD		− 0.086 ± 0.57	
Mean flow (ml/s)	2.0 ± 0.48		2.0 ± 0.62
Bias ± 1.96SD		− 0.071 ± 0.58	
Mean velocity (cm/s)	28 ± 6.8		29 ± 8.8
Bias ± 1.96SD		− 0.077 ± 14	
Peak velocity (cm/s)	84 ± 25		77 ± 34
Bias ± 1.96SD		7.6 ± 44	

^a^Both M1 branches of the middle cerebral artery (MCA). Direction of positive flow is away from the CoW

#### Cerebral aqueduct

Bland–Altman analysis of the ECG and DUS gated 2D flows analysis show a small bias and a wide limit of agreement: bias ± 1.96SD: 0.61 ± 13.6 μl for the net flow volume per heart beat (Fig. 3, Table 3). For the mean flow, bias ± 1.96SD is 0.90 ± 15 μl/s, for the mean velocity 0.78 ± 5.4 cm/s and for the peak velocity − 0.91 ± 4.7 cm/s. No statistically significant differences in flow and velocity between the ECG and DUS- gated results measures were observed. For example, the net volumes per heart beat were − 3.0 ± 5.8 μl and − 3.6 ± 2.1 μl (*p* = 0.91), mean flow − 3.0 ± 6.1 μl and − 3.9 ± 2.4 μl (*p* = 0.82), mean velocity − 0.078 ± 0.18 cm/s and − 0.16 ± 0.13 (*p* = 0.57) and peak velocity − 9.7 ± 1.9 cm/s vs. − 8.8 ± 2.9 (*p* = 0.25), for ECG and DUS gating, respectively. The net flow volume is defined as the sum of the upwards and downwards slope during one heart beat and reflects the net transport of CSF. Note that for the CA measurements, positive flow is by convention defined as directed cranially, towards the ventricles.

### Pulsation artefacts

Pulsation artefacts were slightly more prominent for the DUS gated scans. Out of 18 2D flow scans, six ECG-gated scans had weak pulsation artefacts while four of the DUS-gated scans showed weak pulsation and four had stronger artefacts.

## Discussion

In this work, we have investigated the sensitivity and robustness of a Doppler ultrasound-based (DUS) cardiac synchronization technique for intracranial flow measurements at 7T. Doppler ultrasound is not affected by the magnetohydrodynamic (MHD) effect, and we found that the DUS-device produces clean trigger signals in the ultra-high field MR environment. This in combination with the simple setup makes DUS a promising technique for cardiac synchronization in ultra-high field MR. However, the autocorrelation implemented in the peak detection algorithm was tuned for application in fetal cardiac MRI and was therefore too restrictive for use in healthy adults, which resulted in missed events. Nevertheless, quantitative flow values from ECG and DUS gated scans agreed well.

### Trigger accuracy and precision

The DUS system can be described as consisting of three functional parts, and false triggers can originate from any of them. First, the sensor must be able to detect the signals. Second, the filtering algorithm should clean up the raw signal and output the pulse forms relevant for the chosen application. Third, peak detection and trigger generation should result in reliable trigger signals. This procedure uses some a priori knowledge; for example, it is not likely that an individual RR-interval differs too much from the mean RR-interval and trigger signals that would correspond to such events can be discarded. In the four subjects with many false negative triggers in the DUS-triggered data, the filtered signal from the DUS device was visually assessed as high quality, and the negative triggers likely originated from too restrictive peak detection algorithms. Ongoing development will fine tune the algorithm to be more robust. The DUS sensor is sensitive to chest motion, and deep breathing can cause false negative triggers. Hardware development, for example using an array of sensors, could address this issue and possibly also allow for motion detection. Together, these causes resulted in the DUS giving about the same total number of false negative triggers as the ECG and the POX. The number of false positive triggers was significantly lower for DUS than for ECG, but not significantly different from POX. This may be explained by the fact that the MHD effect increases the T-wave, which can be misinterpreted as an R-wave in the ECG signal [19, 22]. Since the sensitivity measure contains both false negative and positive triggers, it does not differ significantly between DUS, ECG and POX. False negative and positive triggers influence the acquisition and images differently. While false negative triggers mainly prolong the data acquisition, false positive triggers can introduce image artifacts. The quantitative flow and velocity results do not differ significantly between the synchronization methods, demonstrated by the small Bland–Altman biases (Tables 3 and 4, Figs. 3 and 4), showing that there is no systematic difference between the gating methods. Analyzing the FPT, FNT and sensitivity separately for DUS triggering in systole and diastole, we found no statistically significant differences. This is somewhat unexpected as the systolic signal amplitude is generally lower and consisting of several peaks, and could therefore be expected of having a greater chance of being missed by the peak detection algorithm.

One potential concern could be differing artifact strength from the two methods, for example pulsation artefacts. Several events in the cardiac cycle gives detectable ultrasound signals. The DUS sensor can detect a peak in early diastole corresponding to the rapid filling of the left ventricle, and another originating from myocardial motion and blood flow in systole (Fig. 1b). The diastolic signal is easier to detect, with a larger amplitude and more symmetric appearance, but is delayed about 300 ms from the ECG R-peak and about 150 ms after the systolic DUS wave [15]. When the RR-interval varies, it is mainly the diastolic phase that varies in length [34]. When triggering in early diastole, pulsation artefacts are expected as data acquisition will take place at different distance from the R-peak for different k-space lines. The systolic DUS signal occurs in mid-systole, closer to the ECG-generated triggers at the R-peak. For arterial velocity measurements, gating on the systolic wave is therefore preferred. However, for coronary artery imaging, acquisition takes place in mid-diastole and diastolic gating would be optimal [15]. The relevant peak can be selected by transducer placement and signal filtering [15].

### Comparison to earlier studies

Several techniques have been developed to especially address the challenges of cardiac synchronization in high magnetic fields. The main alternatives to DUS as a replacement of standard ECG are pulse oximetry (POX), acoustic cardiac triggering (ACT) and advanced developments of the ECG technique. These and further techniques are discussed below.

Pulse Oximetry (POX) has the benefit of a very easy set-up, consisting of a finger clip containing a light source with red and IR light sources. However, it is sensitive to hand motion and temperature, and is less precise as the trigger time point is determined when the pulse wave reaches the subjects finger. This takes place 200–300 ms after the RR-peak, in the early diastolic phase, and the variation causes pulsation artefacts. It is also shown that POX triggers have a higher temporal variability than required for high-temporal resolution investigations, which can for example cause image blurring [14, 15, 23].

Certain gradient schemes may affect the POX signal, reducing sensitivity. Flow quantification sequences with their fast gradient switching are therefore often prone to cause POX disturbances [13].

Phonocardiogram or acoustic cardiac triggering (ACT) has the benefit of being immune to MHD effects in the same manner as DUS synchronization. The first heart tone is picked up by an acoustic sensor positioned at the subjects’ chest and acoustic noise from the scanner is removed by low-pass filtering [14, 22]. In a recent study at 7T, 23% of the examinations had to be switched from ACT to POX for technical reasons, suggesting that the ACT technique had a similar failure rate as ECG [35].

Another approach is to explore ECG-based methods that are more robust to MHD-induced signal distortions than current implementations of ECG gating (Vector ECG (VCG) [5]). As examples, Krug et al. presented a strategy based on independent component analysis of signals from a 12-lead ECG set-up in a 7T MR [9] while Gregory et al. [36] introduced a 3D representation of the ECG leads and used cross-correlation between data recorded outside and inside the MR scanner. However, the study by Gregory et al. only contains two volunteers, showing large individual performance variations of the 3D method. Another similar method uses the standard 3-lead ECG setup, and shows that inclusion of data from a training phase outside the scanner increases the success rate [19].

Self-gating is a general term for methods that deduce the trigger signal from the acquired data instead of relying on an external sensor. These methods are based on finding a trigger point from the signal variation in the field of view induced by the periodic flow. Several implementations have been proposed, but are not widely available [16–18]. Self-gating depends on using tailored k-space readout schemes, limiting the flexibility of sequence design and requiring non-standard reconstruction algorithms. An extension of the notion of self-gating has been presented [20, 21, 37], where the authors show that the parallel transmit (pTx) coil scattering matrix reflects the heart beat as well as respiration, and can be used for synchronization.

The use of photoplethysmography, an optical technique to detect blood volume changes in the skin, is being explored for many medical applications including bedside detection of cardiac arrhythmias [38] and respiration [39]. One setup for 7T by Spicher et al. [23] using a video camera to record changes in forehead skin tone induced by blood volume changes in the capillary bed was shown to give clear trigger signals. However, this setup was intended for cardiac imaging, and would be difficult to use with a 7T head coil.

Finally, Frauenrath et al. suggested to use the characteristics of the increased MHD effect for gating [6] and several studies have investigated measuring and modelling of the MHD effects throughout the body [40, 41]. These methods show promise, but are at an early stage of technical development.

### Limitations

The main limitation of this study is the low number of subjects, and the fact that all subjects are healthy volunteers. However, as the volunteers were both male and female and had a range of resting heart rates, and each subject had three quantitative scans for each synchronization method, therefore even this small sample gives a good indication of the potential value of the technique.

The interpretation of the trigger data would have been simplified if DUS and ECG trigger signals were recorded simultaneously. However, the equipment available at the time of this study did not allow this. For upcoming studies, the equipment will be improved to allow for simultaneous ECG and DUS recording.

## Conclusions

Doppler ultrasound (DUS) has the ability to produce trigger signals from cardiac motion that are unaffected by the magnetohydrodynamic effects that degrade electrocardiogram (ECG) quality at 7T. In combination with the simple setup, this makes DUS a promising technique for cardiac synchronization in ultra-high field MR quantification of intracranial flow of blood and cerebrospinal fluid. Further development is needed for fine-tuning of the peak detection algorithm which translates the DUS signals to triggers for the MR system.

## Supplementary information

**Additional file 1**. The results of the trigger event analysis when separating the data triggered on the DUS diastolic and systolic signals. There are only four data points for diastolic triggering and five for the systolic triggering, but with this limited amount of data, there is no difference between the two trigger points.

## Data Availability

The datasets and/or analyzed data from the current study can be made available from the author on reasonable request.

## Abbreviations

ACT: Acoustic cardiac triggering
CA: Cerebral aqueduct
CoW: Circle of willis
DUS: Doppler ultrasound
ECG: Echocardiography
fMRI: Functional magnetic resonance imaging
FNT: False negative triggers
FPT: False positive triggers
IR: Infrared
M1: The sphenoidal/horizontal segment of the MCA
MCA: Middle cerebral artery
MHD: Magnetohydrodynamic effect
MR: Magnetic resonance
MRI: Magnetic resonance imaging
RF: Radiofrequency
POX: Pulse oximetry
VCG: Vector electrocardiography
