# Supplementary figures and images for: Doppler ultrasound cardiac gating of intracranial flow at 7T
Source: BMC Med Imaging. 2020 Dec 9;20:128. doi: 10.1186/s12880-020-00523-x (PMC7724705; doi:10.1186/s12880-020-00523-x)

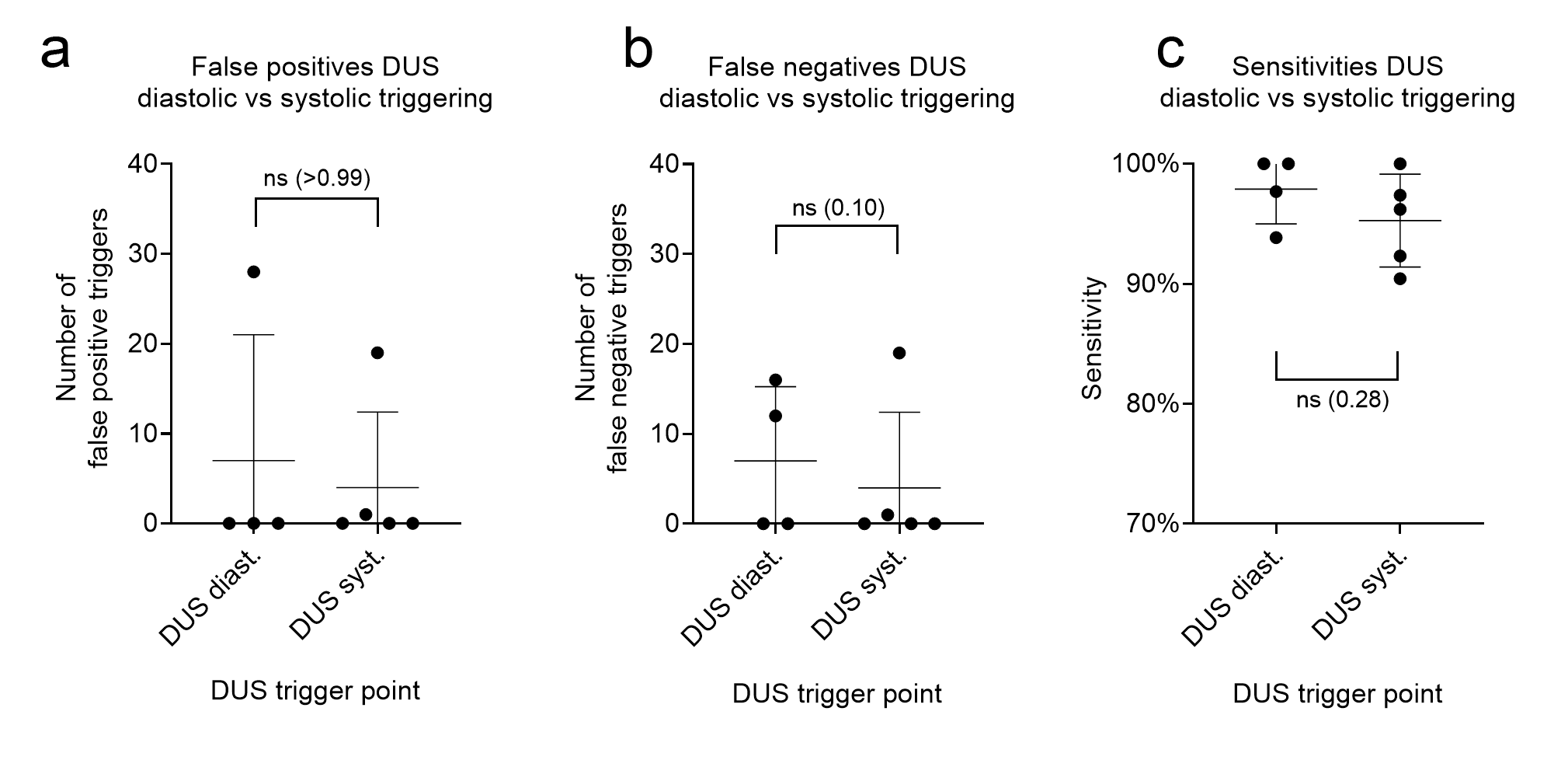

Supplement: Supplementary file 1 — Additional file 1. The results of the trigger event analysis when separating the data triggered on the DUS diastolic and systolic signals. There are only four data points for diastolic triggering and five for the systolic triggering, but with this limited amount of data, there is no difference between the two trigger points. [file 12880_2020_523_MOESM1_ESM.tif]
